# HFO-LADRC Lateral Motion Controller for Autonomous Road Sweeper

**DOI:** 10.3390/s20082274

**Published:** 2020-04-16

**Authors:** Dequan Zeng, Zhuoping Yu, Lu Xiong, Zhiqiang Fu, Zhuoren Li, Peizhi Zhang, Bo Leng, Fengwu Shan

**Affiliations:** 1 School of Automotive Studies, Tongji University, Shanghai 201804, China; zdq1610849@126.com (D.Z.); yuzhuoping@tongji.edu.cn (Z.Y.); fu0zhiqiang@163.com (Z.F.); 1453411@tongji.edu.cn (Z.L.); zhangpeizhitom@126.com (P.Z.); lb9161@163.com (B.L.); shanfw@126.com (F.S.); 2 Clean Energy Automotive Engineering Centre, Tongji University, Shanghai 201804, China; 3 Jiangling Motor Group New Energy Vehicle Co., Ltd., Nanchang 330000, China

**Keywords:** lateral motion control, linear active disturbance rejective control, system uncertainty, robustness

## Abstract

How to make a controller robust and stable to reject the disturbance of uncertainty is an inevitable challenge. Aiming at addressing the lateral control problem for an autonomous road sweeper, a heading-error-based first order linear active disturbance rejective controller (HFO-LADRC) is proposed in this paper. To eliminate the lateral error and the heading error at the same time, a new model, called the heading-error-based model, is proposed for lateral motion, and the Lyapunov function was employed to explore the convergence ability of the heading error and lateral error. Since the heading-error-based model is first order, the ADRC is designed as first order and linear, and each module of the HFO-LADRC has been devised in detail. To ensure solution accuracy, the fourth order Runge–Kutta method was adopted as the differential system solver, and a typical ring scenario and a double lane-changing scenario were designed referencing the standard. Considering the obvious influence, wheelbase uncertainty, steering ratio uncertainty and Gaussian white noise disturbance were taken into account for the tests. The results illustrate that, in the case of both wheelbase uncertainty and steer ratio uncertainty, the HFO-LADRC has strong robustness and stability compared with a typical pure pursuit controller and classical SO-LADRC.

## 1. Introduction

Driven by the labor shortage, the need for safe production processes, and people’s demand for a comfortable life, automation technology has gradually penetrated every aspect of production and life in recent decades [1,2]. For example, automated guided vehicles (AGV) have been broadly applied to the handling of heavy objects or large devices in production environments [3], multi-axis CNC machines have been extensively used to machine parts with sculptured surfaces in the fields of machinofacture and aerospace [4], robots have been developed for welding, spraying and assembling in smart factories [5], firefighting robots have come into service in order to reduce the risks that human firefighters face [6], the Roomba promises its customers “effortless cleaning” or robot vacuums allow owners to “clean your floors without lifting a finger” [7], and feeding assistive robots have been proposed to help older people and people with disabilities in their daily life [8]. Thanks to the development of artificial intelligence [9], machine vision [10], sensor technology [11], etc., autonomous driving technology [12], as a new automation technology which is deeply integrated with various advanced technologies [13], is witnessed to have leapt forward in the last decade [14], and as a result, many unicorn enterprises are known to have developed, such as Waymo [15], Momenta [16], and Apollo [17].

Despite the abundant support of advanced technologies, many problems remain in achieving fully safe autonomous driving [2]. One of the problems to be solved is how to achieve the robustness and stability of motion control, since motion controllers have a core role in generating action commands to stabilize to the reference path or trajectory in the presence of modeling error and other forms of uncertainty [18]. Regarding the tasks to be achieved [19], motion control can be divided into longitudinal motion control [20] and lateral motion control [21]. A longitudinal motion controller is responsible for achieving stable speed tracking and controlling the vehicle to cruise at a predetermined speed or keep a certain distance from the dynamic target in front [22,23,24]. A lateral motion controller has the responsibility to stably and robustly track a reference path, that is, to control the vehicle along the planned path and ensure the vehicle’s safety, ride stability, and ride comfort [25,26,27].

Since the work speed of an autonomous road sweeper is constant at 5 km/h, the only challenge for motion control is how to achieve robust and stable lateral control, that is, how to better track the reference path or trajectory. In order to overcome this practical hurdle, a heading-error-based first order linear active disturbance rejective controller (HFO-LADRC) is proposed in this paper. The main contributions of this manuscript are as follows: (1) A new lateral error model is proposed according to the kinematic relationship of the preview node, fixed onto the vehicle body, and the track node, located in the reference trajectory, in order to describe the lateral motion of an autonomous road sweeper. The model shows that the system is underactuated. (2) A new model, called a heading-error-based model, is proposed for lateral motion, in order to eliminate lateral error and heading error at the same time, and the characteristics of the Lyapunov function prove that the heading-error-based model has the ability to converge both lateral error and heading error. (3) An active disturbance rejective controller (ADRC) is proposed as first order and linear, since the heading-error-based model is first order, and each module of the HFO-LADRC has been devised in detail, including a first-order linear tracking differentiator (LTD), linear extended state observer (LESO), and linear state error feedback (LSEF).

The rest of this paper is organized as follows. Section 2 discusses state-of-the-art technology in related works. Section 3 establishes the lateral error model. Section 4 presents the heading-error-based model, verifies the convergence ability of directional and lateral errors, and presents the design for the HFO-LADRC lateral motion controller. Section 5 selects uncertainty factors, presents simulation scenarios and analyzes the results to validate the controller performance. Section 6 presents the conclusions of the evaluation and suggestions for future works.

## 2. Related Work

The efforts made aimed at conquering the challenge for motion control of autonomous driving systems have paid off. A linear quadratic regulator (LQR) has been used to design a controller for autonomous vehicles in the steady state [28,29]. Alcala et al. [30] proposed an LQR controller, which shows more stable performance than PI and PD control in terms of the ability of an LQR to suppress overshoot. Chuan et al. [31] constrained the sideslip angle in the reasonable region by using a robust LQR controller, which maintains the vehicle stability through the path following process. Literature [32] proposed an iterative algorithm, which relies on an LQR-LMI based strategy using an LPV representation of the closed loop kinematic error model, to adjust the parameters of the non-linear controller to achieve not only stability but also performance specifications. However, one fatal flaw in the LQR is that the algorithm is suitable for linear systems but is not convenient for nonlinear systems, except for making some hypotheses and even instability in high maneuver treatments [33,34]. As one of the traditional strategies that can be used independently of the accurate system model, proportion-integration-differentiation (PID) controllers are very prevalent [35,36]. Pouria et al. embedded adaptive control with anti-windup compensators with a PID controller to conquer the challenge of model uncertainty and saturation in actuators [37]. Since tuning of PID parameters is important because these parameters have a great effect on the stability and performance of the control system, Ankush et al. [38] proposed a harmonic-search-algorithm (HSA) technique to tune the PID parameters. Similarly, as traditional PID fails to ensure lateral control stability due to the inflexible parameter settings, BP-PID (back-propagation proportion-integration-differentiation) was designed by Han et al. [39]. As a prevalent variant of PID, pure pursuit has proven to be an indispensable method for vehicle control owing to its simple implementation and satisfactory performance [16,40]. Snider et al. [41] conducted pure pursuit experiments on the lane change course, which shows that a short look-ahead distance provides more accurate tracking, while a longer distance provides smoother tracking. However, the pure pursuit controller becomes unstable when the lateral error is large (if the look-ahead distance is not increased adaptively and reduced slowly as the vehicle converges to the path) [42]. As another traditional control strategy that is not restricted by the system model, fuzzy control has found many applications in machine intelligence and behavior-based control methods [43,44]. Since fuzzy control has emerged as one of the most active areas for research in dealing with nonlinear control problems and has made many achievements, a fuzzy logic controller was designed for the road following of an autonomous land vehicle by Wu et al. [45]. An adaptive control law for the fuzzy controller is presented to effectively compensate the hydrodynamic effects. Taking into account the uncertainty of environmental interference, it is difficult to establish an accurate motion model [46]. As mentioned by Naranjo et al. [47], the main reason for using the fuzzy approach is that a suitable driving process model is essential for automatic steering wheel control, and, nevertheless, classical approaches frequently fail to yield appropriate models of complex (nonlinear, time-varying, ill-defined) processes—and driving a car certainly falls into this category—whereas fuzzy-logic-based control methods provide an alternative tool for dealing with car and subsystem complexity. However, if the constructions of the rules and membership functions are very complicated, the empirical approach is not only time consuming and labor intensive, but also cannot ensure that the optimum rules and the membership functions are found [48,49]. In order to achieve a model that can tolerate imprecision and has controller parameters that are easy to set at the same time, model predictive control (MPC) [50,51] is one of promising options. To achieve the desired performance during high-speed driving, the MPC controller architecture considers both the kinematic and the dynamic control in a cascade structure [52]. An improved MPC controller based on fuzzy adaptive weight control is proposed to solve the problem of autonomous vehicles in the process of path tracking, which not only ensures tracking accuracy, but also considers vehicle dynamic stability in the process of tracking, and moreover, the problem of driving comfort caused by the application of a classical MPC controller when the vehicle is deviated from the target path is solved [53]. Sun et al. [54] present an MPC path-tracking controller with switched tracking error, which reduces the lateral tracking deviation and maintains vehicle stability for both normal and high-speed conditions. However, MPC is complicated in calculation, and the real-time performance of the algorithm has been challenged [55]. As another promising option, active disturbance rejective control (ADRC) [56,57] is a kind of lightweight calculation with a simple design and anti-interference strategy. The first discussion of ADRCs was presented by Han [58] and was elaborated upon by Han et al. [59,60]. It is a practical control technology whose basic idea is “To reject disturbance with estimating disturbance” [61,62,63,64]. Research conducted by Yang et al. [62] shows that the designed linear active disturbance rejection control could overcome the first order time delay, and it could cope with the difficulties of the presence of parameter uncertainties when dealing with large lag process [63]. Furthermore, Jin et al. [64] employed a small gain theorem to interpret the stability and robustness of linear active disturbance rejection control and it found that linear extended state observer gain, ***w_o_***, and linear state error feedback gain, ***w_c_***, are the only two parameters related to the stability of the controller, and they have a certain proportion to make the system robust.

## 3. Lateral Error Model

**Figure 1:**
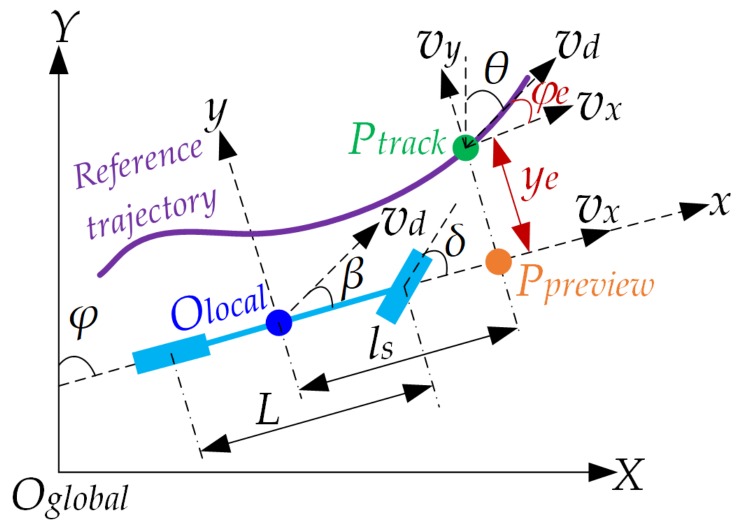
Trajectory tracking diagram.

As shown in Figure 1, the preview node *P_preview_* (the yellow node), which is fixed with the vehicle body and looked as a point of the body, is a forward node with longitudinal distance *l_s_* from the vehicle’s center (the node *O_local_*). The track node *P_track_* (the green node), located in the given reference trajectory (the purple curve), produces the preview node *P_preview_* by projecting in the longitudinal direction *O_local_x*. *y_e_* is the heading angle of the preview node *P_preview_*, *θ* is the heading angle of the track node *P_track_*, *y_e_* is the lateral error from track node *P_track_* to preview node *P_preview_*, and *φ_e_* is the heading error of from track node *P_track_* to preview node *P_preview_*.

The kinematic model between the track node *P_track_* and preview node *P_preview_* is

(1) $${\dot{y}}_{e}={\dot{y}}_{track}-{\dot{y}}_{preview}$$

where, *y_track_* is the lateral position of the track node *P_track_* in vehicle coordinate *O_local_xy*, and *y_preview_* is the lateral position of the preview node *P_preview_* in vehicle coordinate *O_car_xy*.

Since the vehicle could be thought of as a rigid body, there are kinematic relations in the two-dimensional space plane as follows:

(2) $$\left\{\begin{array}{l} {\dot{y}}_{track}=v_{d}\sin\left(\theta -\varphi \right)=v_{d}\sin\varphi_{e}\\ {\dot{y}}_{preview}=v_{d}\sin\beta +l_{s}\dot{\varphi } \end{array}\right.$$

where *v_d_* is the desired speed of vehicle and *β* is the sideslip angle of the vehicle in vehicle coordinate *O_local_xy*.

Since the work speed of an autonomous road sweeper does not exceed 5 km/h, it is a practical assumption that the vehicle has no sideslip, that is, the sideslip angle is *β* ≈ 0. Finally, the lateral error model is obtained as described in Equation (3), which combines Equation (2), Equation (1) and the Ackermann steering formula [52]:

(3) $${\dot{y}}_{e}=v_{d}\sin\varphi_{e}-l_{s}\dot{\varphi }=v_{d}\sin\varphi_{e}-l_{s}w_{s}=v_{d}\sin\varphi_{e}-\frac{l_{s}}{L}v_{d}\tan\delta$$

where *w_s_* is the derivative of the heading angle *φ*, which represents the yaw velocity of the vehicle, *L* is the wheelbase, and *δ* is the steering angle.

## 4. HFO-LADRC Controller Design

### 4.1. Heading-Error-Based Model

Equation (3) is the lateral-error-based format system model, and the system model shows that lateral error and heading error are coupled, which also shows that the system is an underactuated model. In order to eliminate the lateral error and the heading error at the same time, for lateral motion, a new heading-error-based model is proposed [65,66]:

(4) $$z=c_{0}\tanh\left(c_{1}y_{e}\right)+c_{2}\varphi_{e}$$

where *z* is the new format heading error, and the constant factors are *c*_0_ > 0, *c*_1_ > 0, and *c*_2_ > 0.

In order to prove that the system has the ability to converge both lateral error and heading error, the Lyapunov function is designed as follows [65,66]:

(5) $$V=\frac{1}{2}y_{e}^{2}$$

Then, the derivative of the Lyapunov function is

(6) $$\dot{V}=y_{e}{\dot{y}}_{e}=y_{e}\left(v_{d}\sin\varphi_{e}-l_{s}\dot{\varphi }\right)$$

Considering the desired speed to be slow, it is a practical assumption that the speed of the circular motion is negligible, that is, $\dot{\varphi }$ ≈ 0, and combining Equation (3) with Equation (6) gives

(7) $$\dot{V}=y_{e}v_{d}\sin\left\{\frac{1}{c_{2}}\left[z-c_{0}\tanh\left(c_{1}y_{e}\right)\right]\right\}$$

If *z* = 0, there is [65,66]

(8) $$\dot{V}=y_{e}v_{d}\sin\left[-\frac{c_{0}}{c_{2}}\tanh\left(c_{1}y_{e}\right)\right]$$

If we make 0 < *c*_0_/*c*_2_ < *π*, there are

(1) when *y_e_* > 0, there is [65,66]
  (9) $$\sin\left[-\frac{c_{0}}{c_{2}}\tanh\left(c_{1}y_{e}\right)\right]<0\Rightarrow \dot{V}<0,V>0$$
(2) when *y_e_* < 0, there is [65,66]
  (10) $$\sin\left[-\frac{c_{0}}{c_{2}}\tanh\left(c_{1}y_{e}\right)\right]>0\Rightarrow \dot{V}<0,V>0$$
(3) when *y_e_* ≡ 0, there is
  (11) $$\dot{V}\equiv 0,V\equiv 0$$

Therefore, the Lyapunov function has the following characteristics:

(12) $$\dot{V}\leq 0,V\geq 0$$

Finally, the characteristics means that when *z* converges to 0, both *y_e_* and *φ_e_* converge to 0.

### 4.2. Controller Design

More generally, when considering the uncertainty of the system, as there are inevitable uncertainties, such as external interference, vehicle speed error, observation noise of steering angle, etc., the differential format model for the heading-error-based system is as follows:

(13) $$\left\{\begin{array}{l} \dot{z}=\dot{f}\left(v,\theta ,\varphi ,L,l_{s},\delta ,w\right)-c_{2}{\dot{\varphi }}_{e}=\dot{f}\left(v,\theta ,\varphi ,L,l_{s},\delta ,w\right)+b_{0}u\\ b_{0}=-c_{2}\frac{v_{d}}{L}\\ u=\tan\delta \end{array}\right.$$

where *f*(*v*, *θ*, *φ*, *L*, *l_s_*, *δ*, *w*) is the uncertainty part of system.

**Figure 2:**
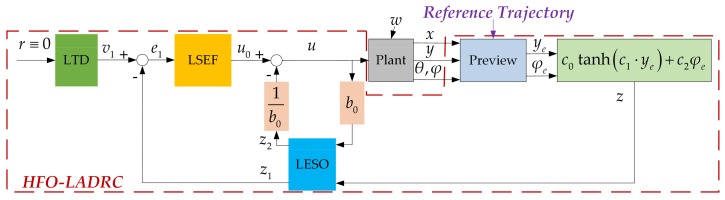
Heading-error-based first order linear active disturbance rejective controller (HFO-LADRC) control scheme.

Correspondingly, the heading-error-based first order linear active disturbance rejection controller (HFO-LADRC) is designed as in Figure 2, where the LTD is the linear tracking differentiator, LESO is the linear extended state observer, LSEF is linear state error feedback, Plant is the vehicle steer system and Preview is the lateral error model, which tracks the reference trajectory to calculate lateral error *y_e_* and heading error *φ_e_*.

Since the control target is making heading error *z* = 0, the reference input of LTD *r* ≡ 0 as first-ordered system, that is, the LTD output *v*_1_ at discrete time scale *k* + 1 is [60,61]

(14) $$v_{1}\left(k+1\right)\equiv 0$$

According to Equation (13), the state space format of the heading-error-based system model is defined as follows:

(15) $$\left\{\begin{array}{l} {\dot{x}}_{1}=x_{2}+b_{0}u=\dot{z}\\ x_{2}=f\left(v,\theta ,\varphi ,L,l_{s},\delta ,w\right)\\ {\dot{x}}_{2}=\dot{f}\left(v,\theta ,\varphi ,L,l_{s},\delta ,w\right)\\ \eta =x_{1} \end{array}\right.$$

where *x*_1_ is *z*, *x*_2_ is the uncertainty part of system, and *η* is the output of the LESO. 

The state observer for Equation (4) to estimate the extended state *x*_2_ in discrete space is [62,63]

(16) $$\left\{\begin{array}{l} e\left(k\right)=z_{1}\left(k\right)-z\left(k\right)\\ z_{1}\left(k+1\right)=z_{1}\left(k\right)+T_{LESO}\left[z_{2}\left(k\right)-2\omega_{o}e\left(k\right)+b_{0}u\left(k\right)\right]\\ z_{2}\left(k+1\right)=z_{2}\left(k\right)+T_{LESO}\left[-\omega_{o}^{2}e\left(k\right)\right] \end{array}\right.$$

where *T_Leso_* is the LESO sampling period, and *w_o_* (>0) is the LESO gain.

Since the HFO-LADRC is first order, the LSEF was designed as proportion controller [63,64]:

(17) $$\left\{\begin{array}{l} e\left(k+1\right)=v_{1}\left(k+1\right)-z_{1}\left(k+1\right)\\ u_{0}\left(k+1\right)=\frac{\omega_{c}}{b_{0}}e\left(k+1\right) \end{array}\right.$$

where *u*_0_ is the virtual control output of the LSEF, and *w_c_* is the LSEF gain.

Finally, synthesis of the control value is deduced as follows [61,62]:

(18) $$u\left(k+1\right)=u_{0}\left(k+1\right)-\frac{z_{2}\left(k+1\right)}{b_{0}}$$

## 5. Experimental Results and Discussion

**Figure 3:**
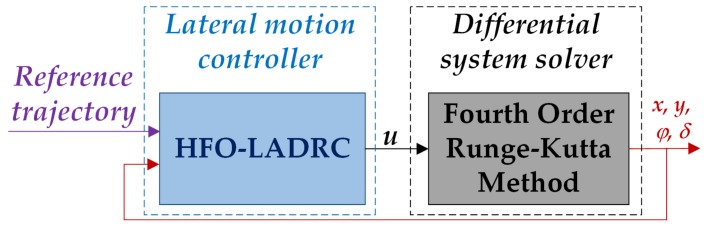
Solving diagram.

Simulations were conducted in a Matlab/Simulink environment with *m* language. The fourth order Runge-Kutta method [67] was adopted as the differential system solver, since the Fourth order Runge-Kutta method has fifth order truncation error [68], which could ensure the solution accuracy. As shown in Figure 3, the HFO-LADRC will generate the steer command *u* by tracking the reference trajectory, and the Fourth order Runge-Kutta method, representing the autonomous road sweeper differential system solver, makes the sweeper move and produce the current state.

In order to make the simulation results closer to the actual conditions, a typical ring scenario and a double-lane collision avoidance scenario were designed, referencing *ISO3888-2:2011* [69]. There was (1) a ring scenario consisting of two 35 m straight lanes plus two semicircles with a radius of 2.5 m and (2) a double-lane scenario consisting of a straight interval 2 m in width and 25 m in length.

Considering the factors that influence the control strategy most in the model [70], the main uncertainty factors lie in the wheelbase and steering ratio. Furthermore, the typical pure pursuit algorithm [40,41,42] and classical second-order LADRC method (SO-LADRC) [70,71,72] were adopted as comparison algorithms. Table 1 lists the key parameters for the autonomous road sweeper and the controller.

### 5.1. Wheelbase Uncertainty

Considering the inevitable processing, assembly error and a series of other disturbance factors, the error of the actual wheelbase can be controlled within 0.1 m. For this reason, the real wheelbase *L* is divided into three grades, which are respectively 1.24 m, 1.34 m, and 1.44 m, since the designed wheelbase *L* is 1.34 m. Therefore, the simulation was conducted in three situations as listed in Table 2.

**Figure 4:**
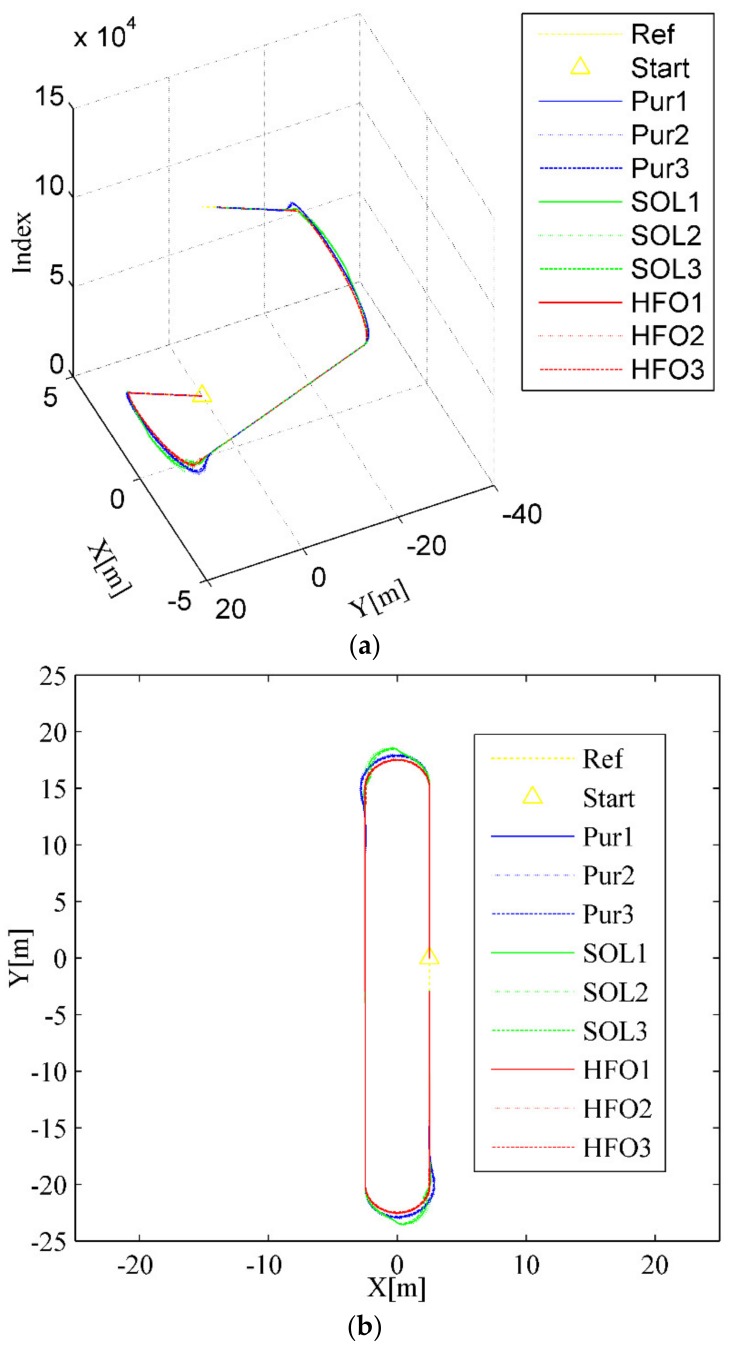

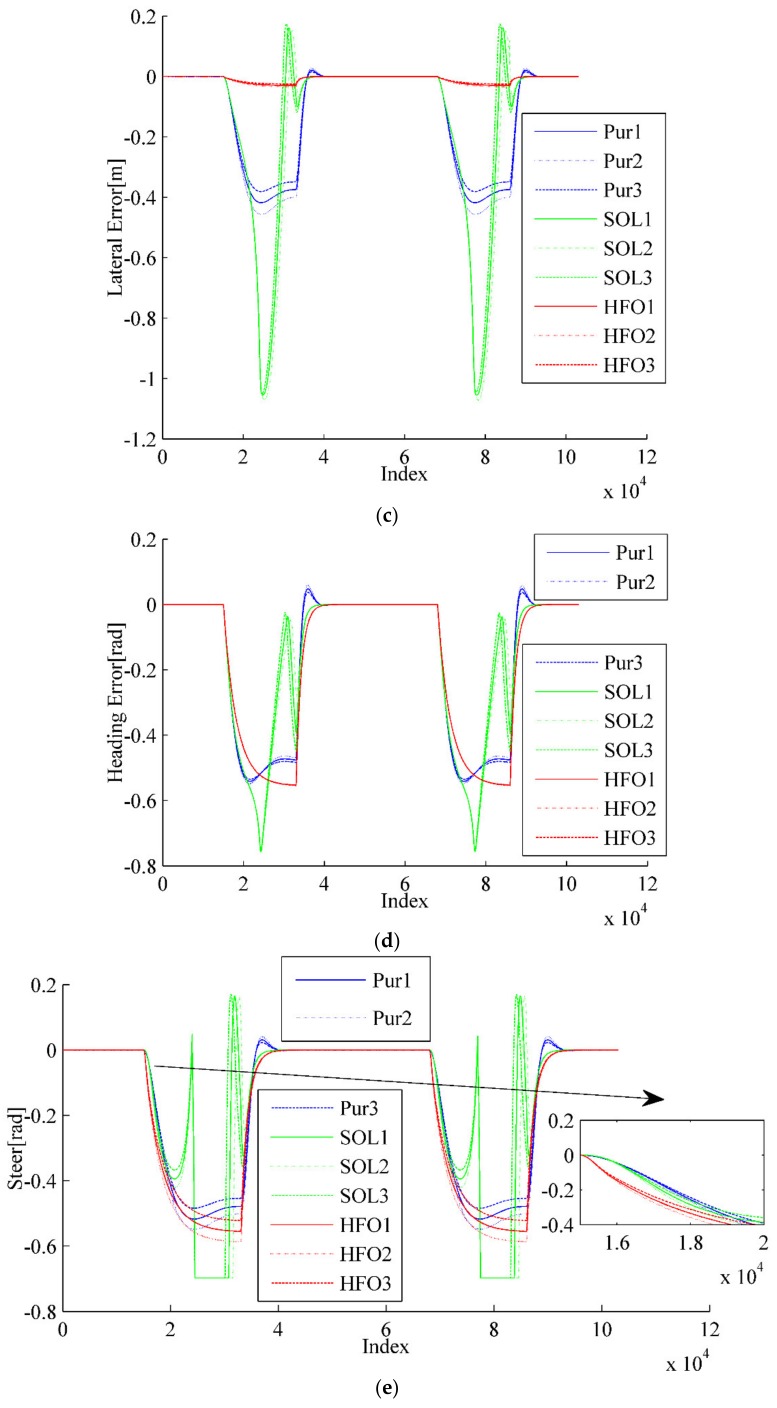
Ring test scenario. (**a**) Trajectory tracking (3D), (**b**) Trajectory tracking (2D), (**c**) Lateral error, (**d**) Heading error, (**e**) Steer angle.

For the ring test scenario, as shown in Figure 4a,b, all three methods can accomplish the task of trajectory tracking; however, the tracking trajectory of the pure pursuit controller (the blue curves) deviates significantly from the arc to the straight compared with that of the SO-LADRC (the green curves) and the HFO-LADRC (the red curves) methods. This characteristic is reflected in the lateral error, which changes from decreasing to increasing and then stabilizes over a period, as shown in Figure 4c,d. However, the most striking phenomenon is that with the SO-LADRC (the green curves), there is a steep increase in the process of entering the arc from the straight, then there is a very obvious shock in the early stage of entering the straight from the arc, and finally it stabilizes in the straight, as depicted in Figure 4c,d. This striking phenomenon comes from the instability of the steering angle, as shown in Figure 4e. Since the SO-LADRC tracks the change in the Y value rather than the lateral error, the Y value of the arc segment is the sine function of the heading angle *θ* (Y is proportional to sin*θ*, which is a function that increases and then decreases with the heading angle *θ*), and the derivative is the cosine function (which can be regarded as the difference of the Y value, and this is a function that decreases and then increases with the heading angle *θ*). Due to the Y increasing with the controller of the SO-LADRC steering wheel but the growth rate of Y going down, the controller has to reduce steer in the process of entering the arc from the straight. Then, to go from the arc into the straight, the controller has to increase the steer angle and then decrease the angle. Finally, the peak error of the SO-LADRC is the largest, reaching almost 1.1 m, whereas the HFO-LADRC has the lowest peak error, reaching 0.0342 m, as shown in Figure 4c. For this reason, compared with the SO-LADRC, the lateral error reduction capacity of the HFO-LADRC is close to two orders of magnitude, and although the error of pure pursuit is also relatively low, the lateral error reduction capacity of the HFO-LADRC is still within one order of magnitude, as the peak lateral error of pure pursuit reaches 0.457 m. The above data further indicate that, in the case of wheelbase uncertainty, the HFO-LADRC has strong robustness and stability compared with pure pursuit and the SO-LADRC in a ring test scenario.

**Figure 5:**
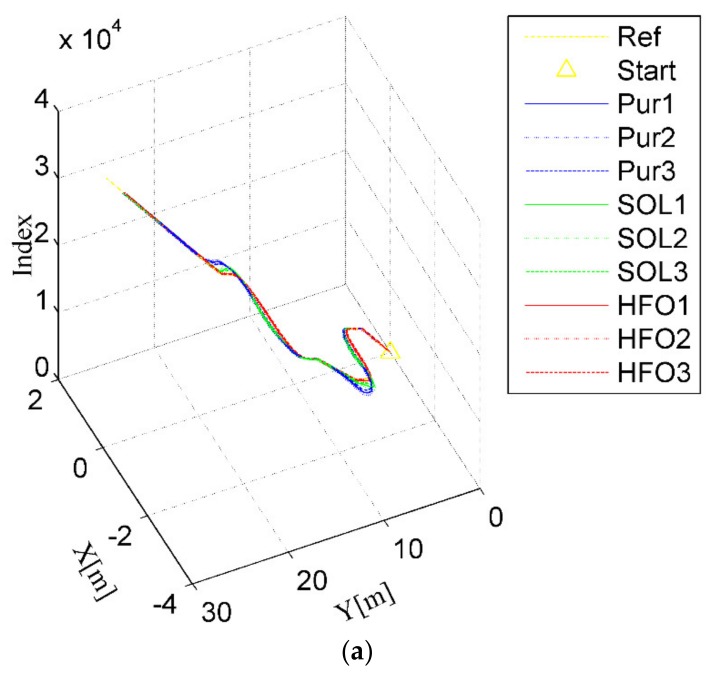

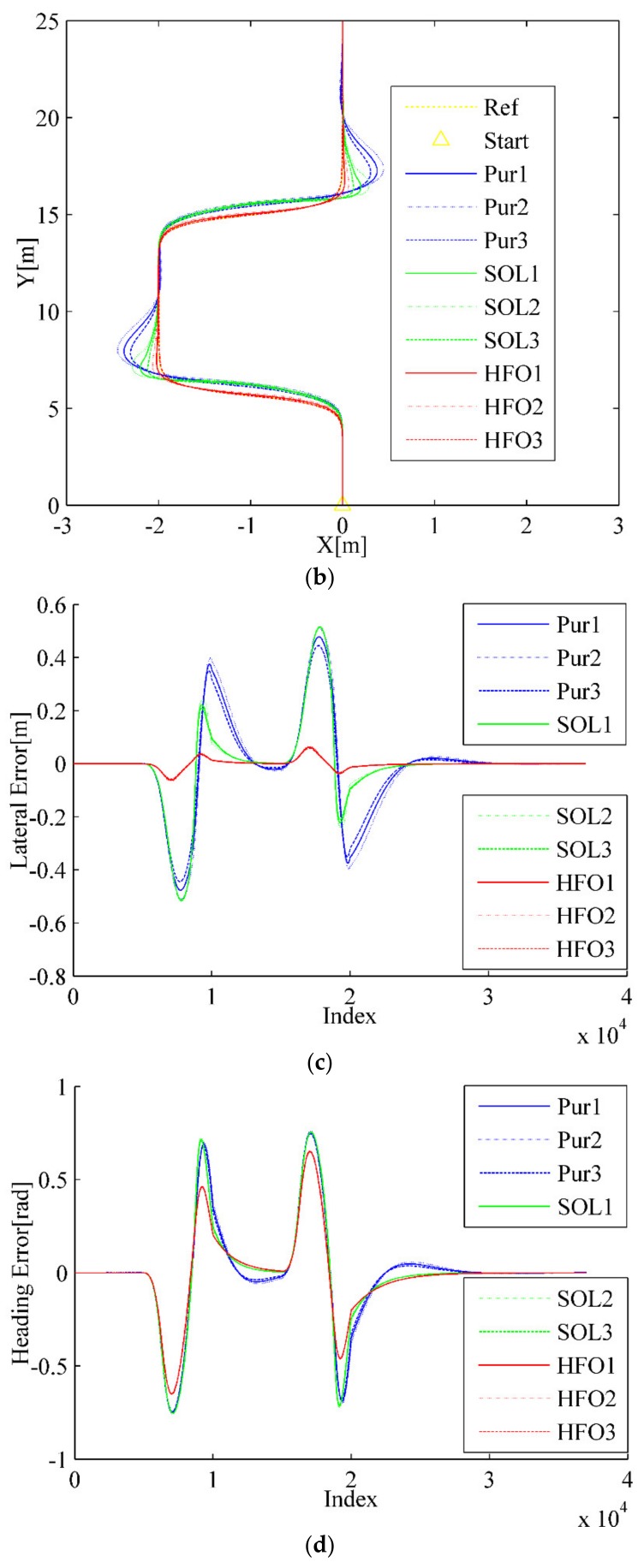

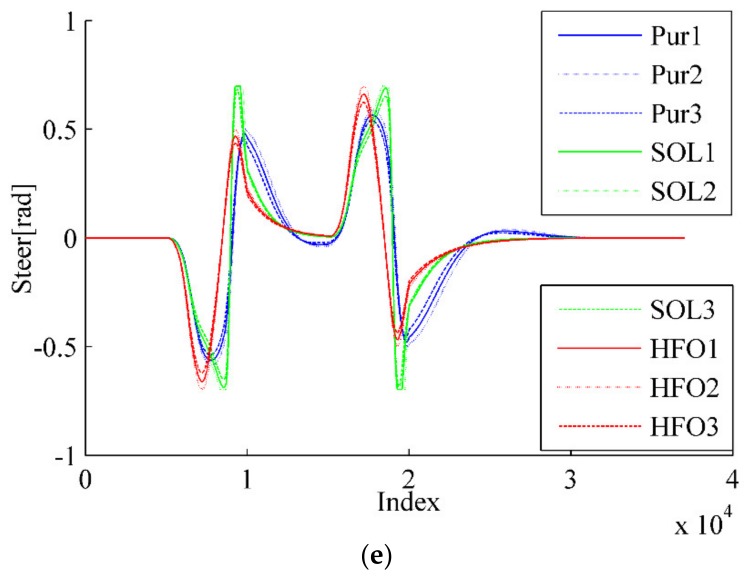
Double lane-changing test scenario. (**a**) Trajectory tracking (3D), (**b**) Trajectory tracking (2D), (**c**) Lateral error, (**d**) Heading angle, (**e**) Steer angle.

Another typical scenario is represented in the double lane-changing obstacle avoidance tracking test, as shown Figure 5. Only pure pursuit (the blue curves) has significant overshoot when lane change is about to occur, as depicted in Figure 5a,b, although all the methods will eventually get the job done. This is evident in the variation of the lateral error for pure pursuit, which always decreases first, then increases, and finally stabilizes when changing lanes into another straight, as shown in Figure 5c,d. Although there is no obvious overshoot with the SO-LADRC (the green curves), the peak error is higher than that in pure pursuit, even if the margin of error is less obvious. The answer lies in the change in the angle of the steering wheel, as shown in Figure 5e, which shows that the steering angle response of the HFO-LADRC is faster than that of the other two methods. Since the change rate of the steering wheel angle in the SO-LADRC has a process of first decreasing and then increasing when the steering wheel angle is close to the peak, this makes the SO-LADRC act a little slower than pure pursuit in error elimination, which results in lateral error being a little higher than that in pure pursuit. It is precisely because the steering angle response of the HFO-LADRC is faster than that of the other two methods that the lateral error of the HFO-LADRC is the lowest, as shown in Figure 5c. Finally, the peak error of the SO-LADRC is the largest, reaching almost 0.5147 m, whereas the HFO-LADRC has the lowest peak error, reaching 0.04649 m, as shown in Figure 5c. For this reason, compared with the SO-LADRC and pure pursuit, the lateral error reduction capacity of the HFO-LADRC is closer to one order of magnitude than the SO-LADRC and pure pursuit, as the peak lateral error of pure pursuit reaches 0.5094 m. The above data further illustrate that, in the case of wheelbase uncertainty, the HFO-LADRC has strong robustness and stability compared with pure pursuit and the SO-LADRC in the double lane-changing obstacle avoidance tracking test scenario.

### 5.2. Steer Ratio Uncertainty

The steer ratio uncertainty is the other key factor, due to the inevitable gear wear, elastic element aging, and a series of other disturbance factors. The error of the actual steer ratio can be controlled within 1.0. Therefore, the real steer ratio *i* is divided into three grades, which are respectively 4.0, 5.0, and 6.0, since the designed steer ratio *i* is 5.0. Then, the simulation is conducted in three situations as listed in Table 3.

**Figure 6:**
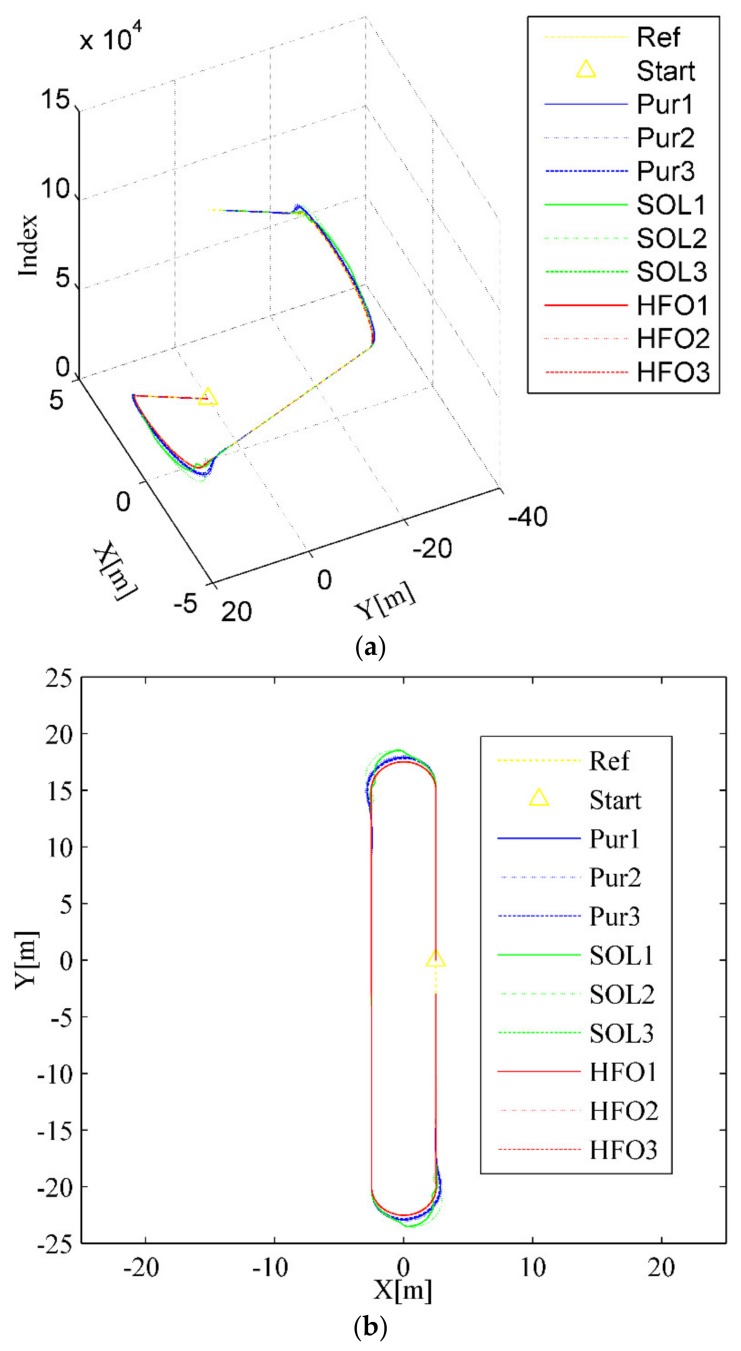

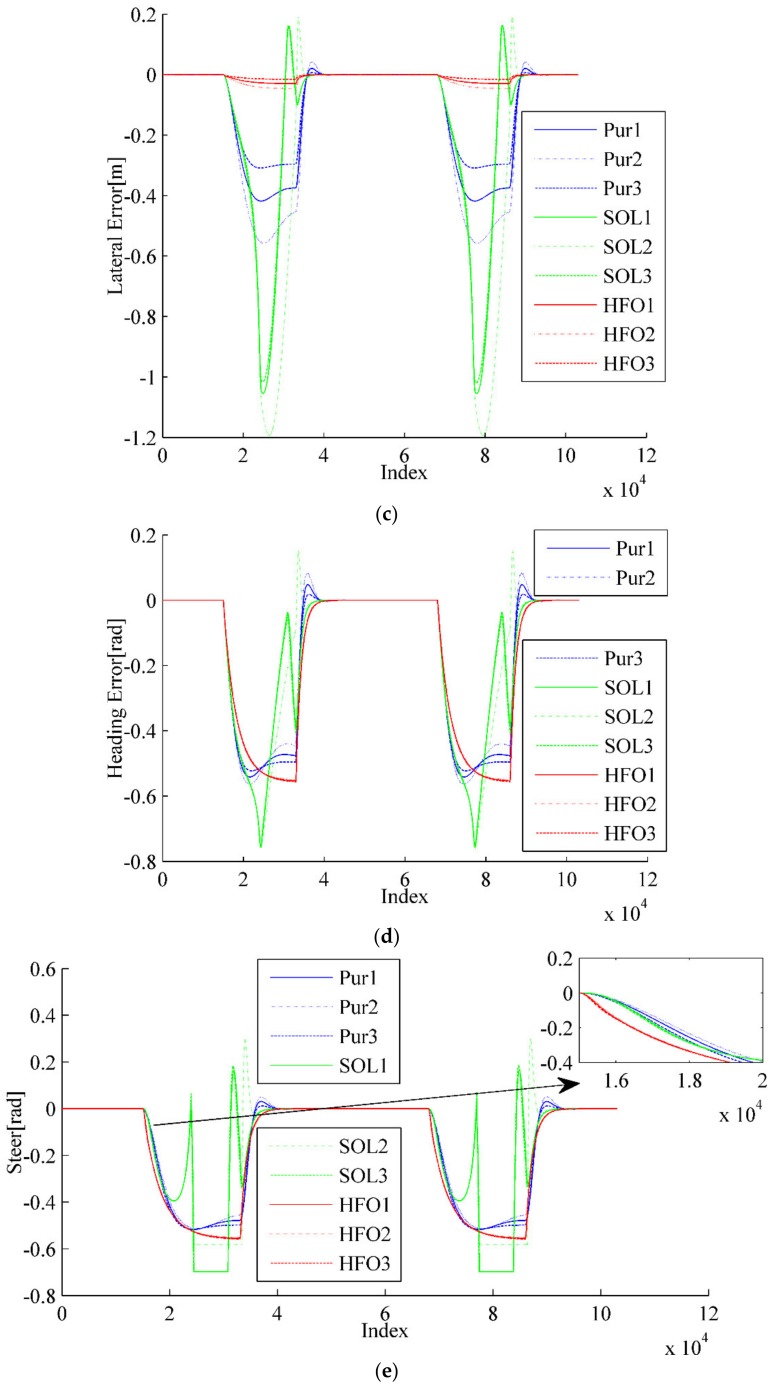
Ring test scenario. (**a**) Trajectory tracking (3D), (**b**) Trajectory tracking (2D), (**c**) Lateral error, (**d**) Heading angle, (**e**) Steer angle.

For the ring test scenario, as shown in Figure 6a,b, all three methods can complete the task of trajectory tracking; however, the tracking trajectory of pure pursuit (the blue curves) deviates significantly from the arc to the straight. With the increase of steer ratio, overshoot will occur for the SO-LADRC (the green curves). This overshoot is directly reflected in the lateral error curve, as shown in Figure 6c,d, which changes from decreasing to increasing, and then stabilizes over a period. However, similar to that in the wheelbase uncertainty test, the most striking phenomenon of the SO-LADRC (the green curves) still exists, as shown in Figure 6e, which shows that the steering angle response of the HFO-LADRC is faster than that of the other two methods. Finally, the peak error of the SO-LADRC is the largest, reaching almost 1.2 m, whereas the HFO-LADRC has the lowest peak error, reaching 0.0462 m, as shown in Figure 6c. For this reason, compared with the SO-LADRC, the lateral error reduction capacity of the HFO-LADRC is close to two orders of magnitude, and although the error of pure pursuit is also relatively low, the lateral error reduction capacity of the HFO-LADRC is still within one order of magnitude, as the peak lateral error of pure pursuit reaches 0.556 m. The above data further indicate that, in the case of steer ratio uncertainty, the HFO-LADRC has strong robustness and stability compared with pure pursuit and the SO-LADRC in a ring test scenario.

**Figure 7:**
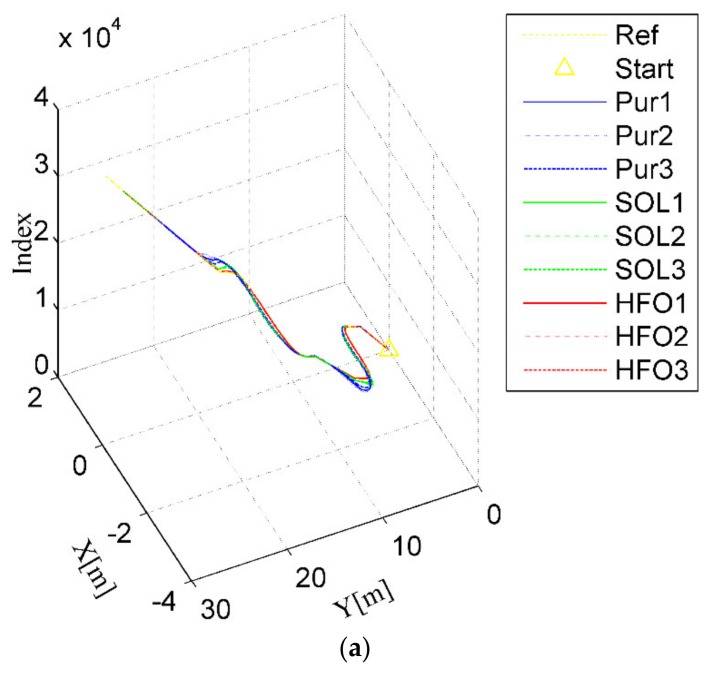

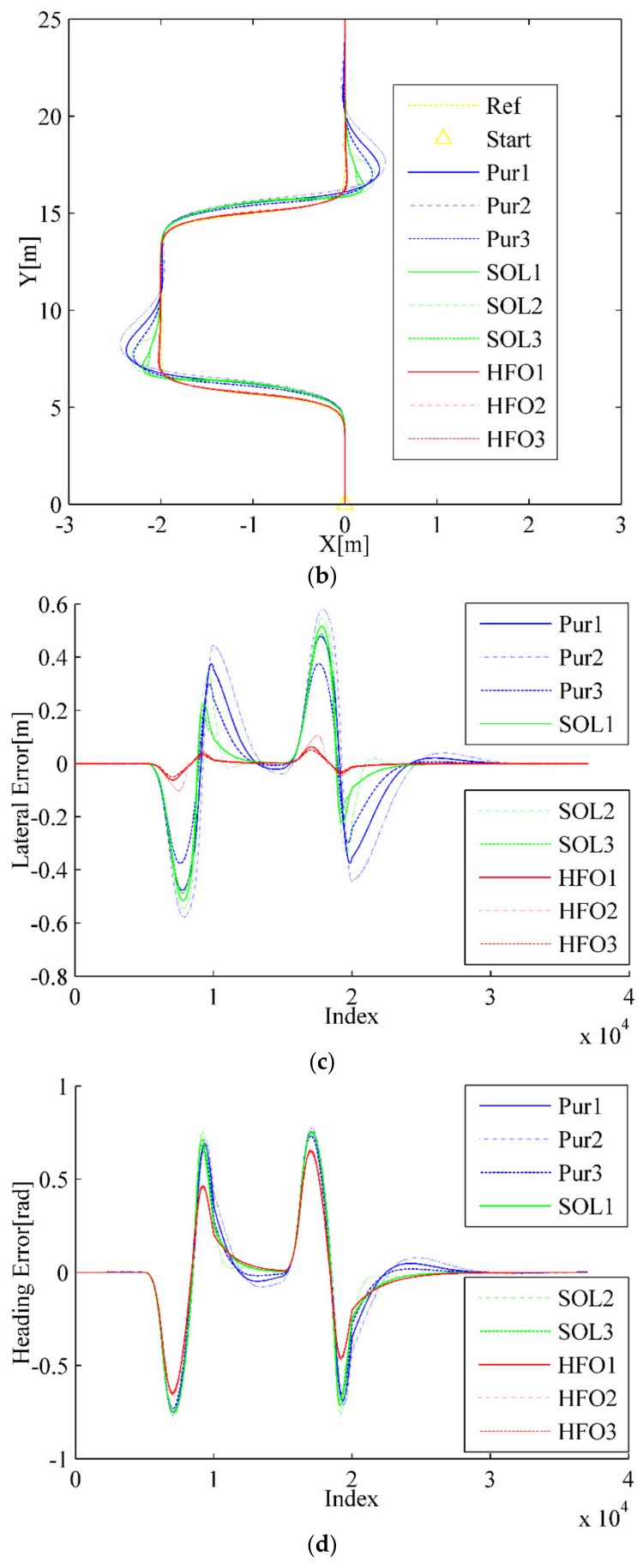

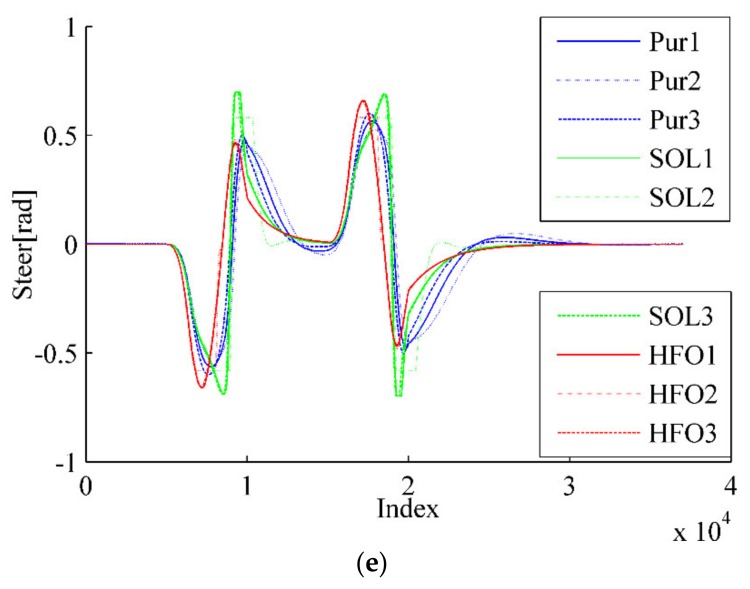
Double lane-changing test scenario. (**a**) Trajectory tracking (3D), (**b**) Trajectory tracking (2D), (**c**) Lateral error, (**d**) Heading angle, (**e**) Steer angle.

A further typical scenario can be seen in the double lane-changing for obstacle avoidance tracking test, as shown Figure 7. Only pure pursuit (the blue curves) has obvious overshoot when lane change is almost accomplished, as illustrated in Figure 7a,b, although all methods will eventually fulfill the tracking task. This is visible in the variation of the lateral error for pure pursuit, which always decreases first, then increases, and finally stabilizes when changing lanes into another straight, as shown in Figure 7c,d. However, although there is no distinct overshoot in the SO-LADRC (the green curves), the peak error is higher than that in pure pursuit. This is consistent with the behavior of wheelbase uncertainty, and the answer lies in the change in the angle of the steering wheel, as shown in Figure 7e. Due to the rate of the steering angle in the SO-LADRC having a process of first decreasing and then increasing when it is close to the peak, it makes the SO-LADRC act a little slower than pure pursuit in error elimination, which leads to lateral error being a little higher than that in pure pursuit. As the steering angle response of the HFO-LADRC is faster than that of the other two methods, the lateral error of the HFO-LADRC is the lowest, as shown in Figure 7c. Finally, the peak error of pure pursuit is the largest, reaching almost 0.5777 m, whereas the HFO-LADRC has the lowest peak error, reaching 0.08752 m, as shown in Figure 7c. For this reason, compared with the SO-LADRC and pure pursuit, the lateral error reduction capacity of the HFO-LADRC is closer to one order of magnitude than the SO-LADRC and pure pursuit, as the peak lateral error of pure pursuit reaches 0.5463 m. The above data further illustrate that, in the case of steer ratio uncertainty, the HFO-LADRC has strong robustness and stability compared with pure pursuit and the SO-LADRC in the double lane-changing obstacle avoidance tracking test scenario.

### 5.3. Gaussian White Noise Disterbance

**Figure 8:**
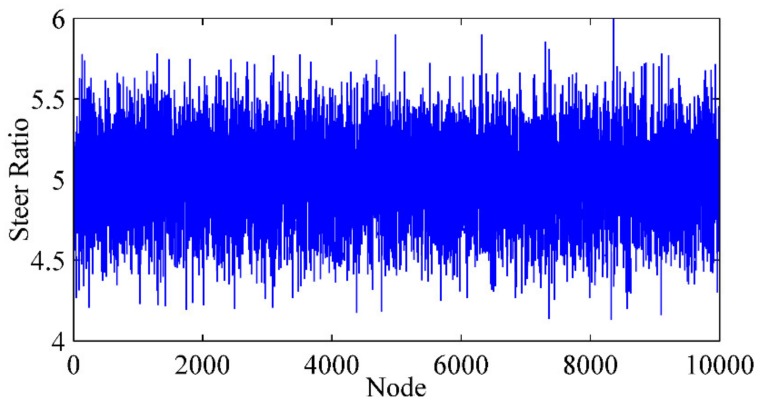
Steer ratio with Gaussian white noise (GWN).

Unlike the wheelbase, the steer ratio could vary during the operation of the vehicle. Therefore, a Gaussian white noise (GWN) disturbance, which has expectation as 5 and mean variance as 0.25, was superimposed on the designed steer ratio to simulate the change, as shown in Figure 8. Then, the simulation was conducted in three situations, as listed in Table 4.

**Figure 9:**
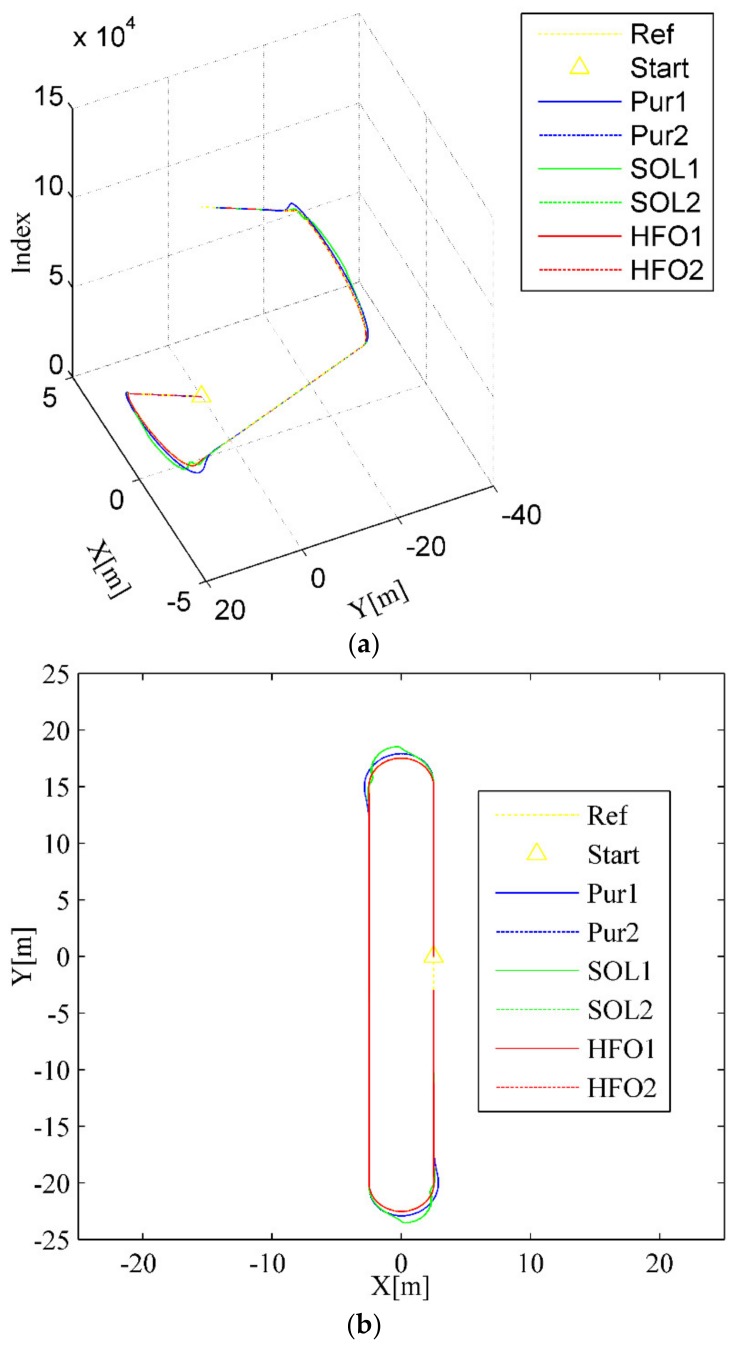

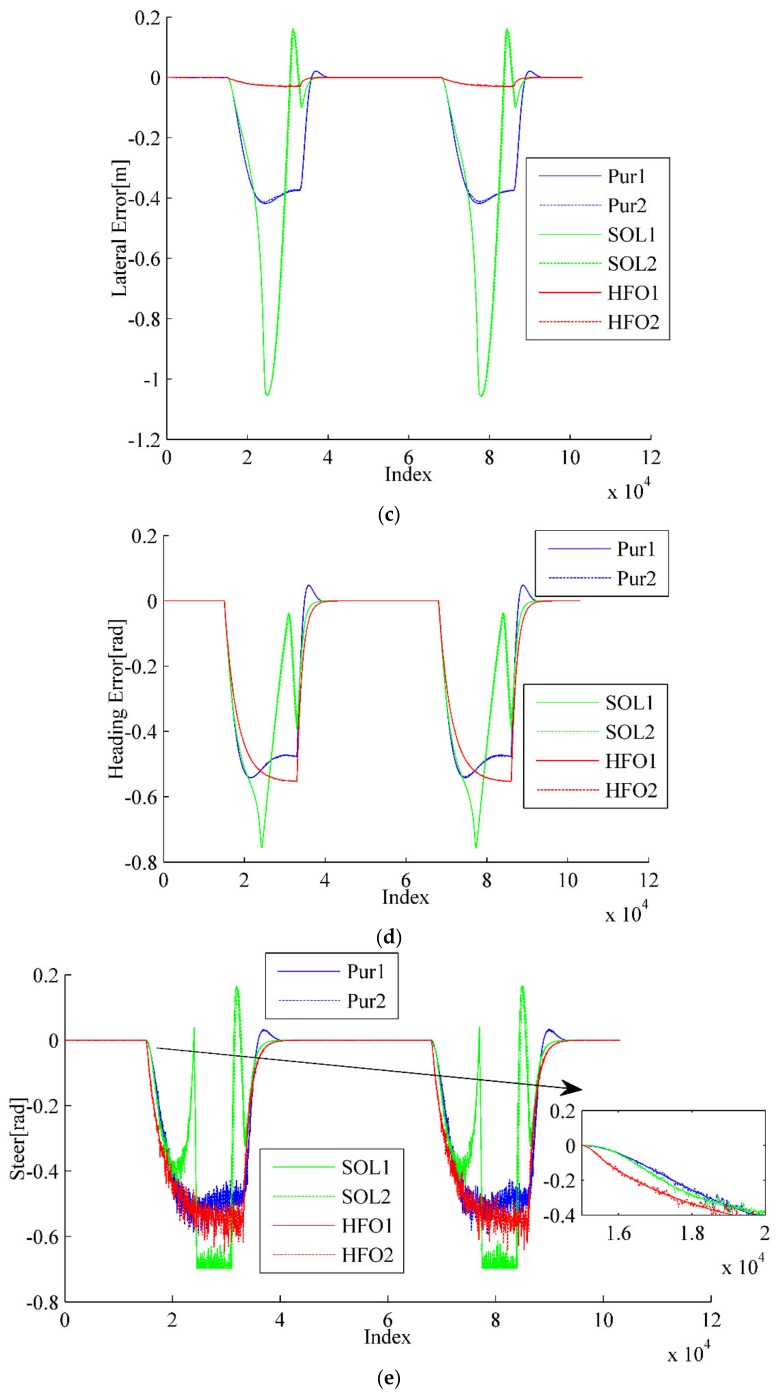
Ring test scenario. (**a**) Trajectory tracking (3D), (**b**) Trajectory tracking (2D), (**c**) Lateral error, (**d**) Heading angle, (**e**) Steer angle.

As shown in Figure 9a,b for ring test scenario, the tracking trajectory of pure pursuit (the blue curves) deviates significantly from the arc to the straight, although the three methods can finish the task of tracking trajectory. Similar with that in the steer ratio uncertainty test, the most striking phenomenon of the SO-LADRC (the green curves) still exists, as shown in Figure 9c,d. This is consistent with the behavior of steer ratio uncertainty, and the answer lies in the change in the angle of the steering wheel, as shown in Figure 9e, which shows that the steering angle response of the HFO-LADRC is faster than that of the other two methods. In addition, the peak lateral error of the SO-LADRC is the largest, reaching almost 1.058 m, whereas the HFO-LADRC has the lowest peak error, reaching 0.031 m, as shown in Figure 9c. For this reason, compared with the SO-LADRC, the lateral error reduction capacity of the HFO-LADRC is close to two orders of magnitude, and although the error of pure pursuit is also relatively low, the lateral error reduction capacity of the HFO-LADRC is still within one order of magnitude, as the peak lateral error of pure pursuit reaches 0.418 m. For heading error, as shown in Figure 9d, the peak heading error of the SO-LADRC is the largest, reaching almost 0.756 rad, where pure pursuit has the lowest peak error, reaching 0.541 rad, and the peak heading error of the HFO-LADRC reaches 0.554 rad. The above data further indicate that, in the case of Gaussian white noise disturbance, the HFO-LADRC has strong robustness and stability compared with pure pursuit and the SO-LADRC in a ring test scenario.

**Figure 10:**
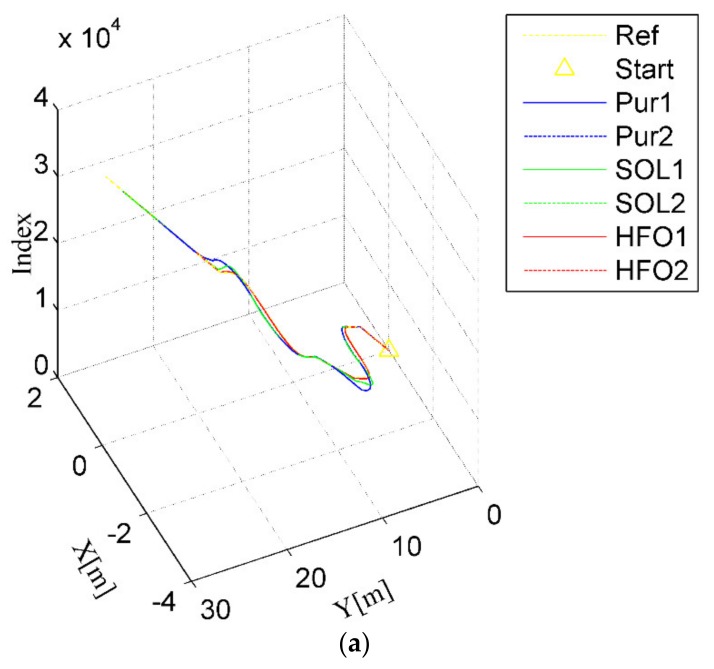

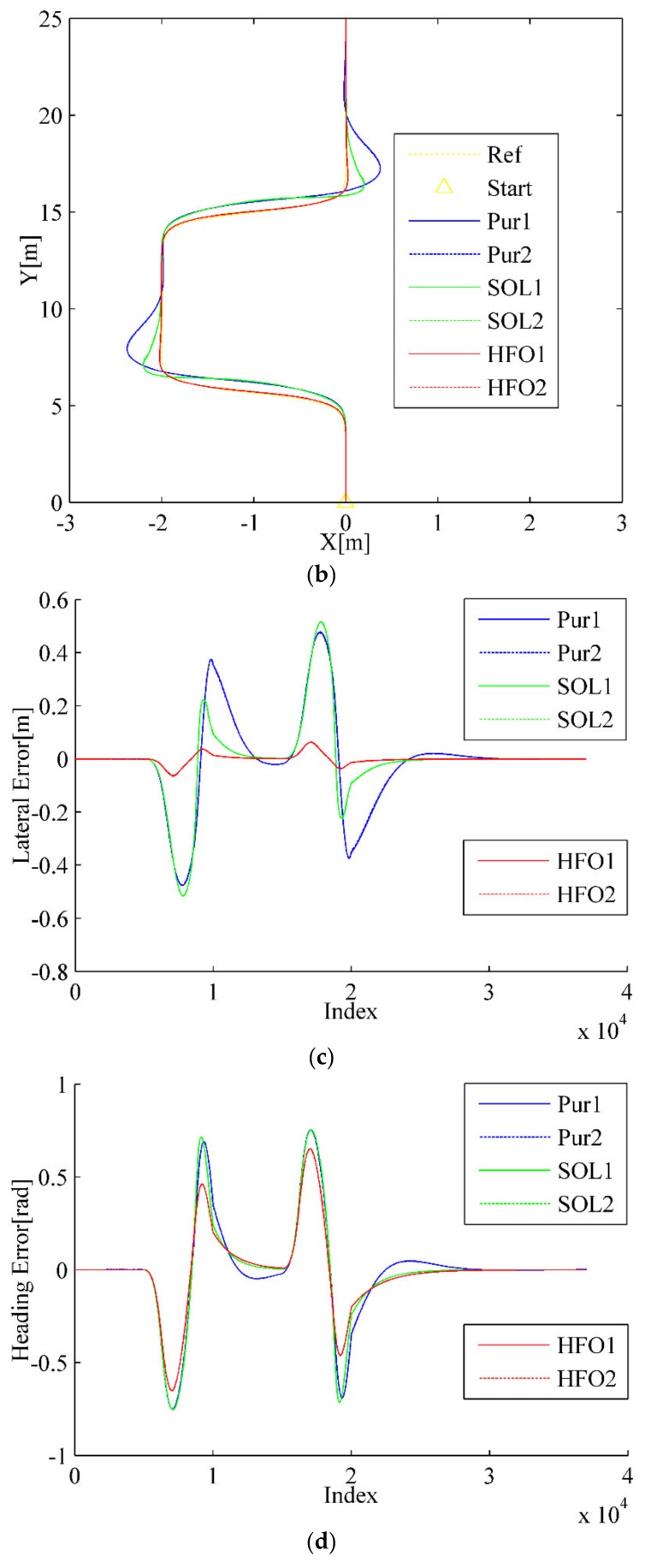

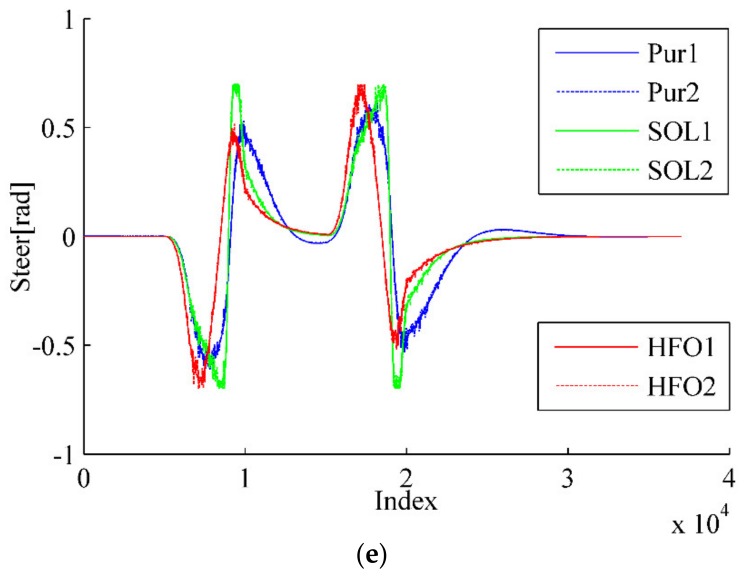
Double lane-changing test scenario. (**a**) Trajectory tracking (3D), (**b**) Trajectory tracking (2D), (**c**) Lateral error, (**d**) Heading angle, (**e**) Steer angle.

Figure 10 is another typical scenario as part of the double lane-changing for obstacle avoidance tracking test. Only pure pursuit (the blue curves) has obvious overshoot when lane change is almost accomplished, as illustrated in Figure 10a,b, although all methods will eventually fulfill the tracking task. This is visible in the variation of the lateral error for pure pursuit, which always decreases first, then increases, and finally stabilizes when changing lanes into another straight, as shown in Figure 10c. However, although there is no distinct overshoot in the SO-LADRC (the green curves), the peak lateral and heading error are higher than that in pure pursuit, as shown in Figure 10c,d. This is consistent with the behavior of wheelbase uncertainty, and the answer lies in the change in the angle of the steering wheel, as shown in Figure 10e. As the steering angle response of the HFO-LADRC is faster than that of the other two methods, as shown in Figure 10e, the lateral error of the HFO-LADRC is the lowest, as shown in Figure 10c. Finally, the peak lateral error of the SO-LADRC is the largest, reaching almost 0.5122 m, whereas the HFO-LADRC has the lowest peak error, reaching 0.058 m, as shown in Figure 10c. For this reason, compared with the SO-LADRC and pure pursuit, the lateral error reduction capacity of the HFO-LADRC is closer to one order of magnitude than the SO-LADRC and pure pursuit, as the peak lateral error of pure pursuit reaches 0.470 m. The above data further illustrate that, in the case of Gaussian white noise disturbance, the HFO-LADRC has strong robustness and stability compared with pure pursuit and the SO-LADRC, in the double lane-changing obstacle avoidance tracking test scenario.

## 6. Conclusions

Aiming at satisfying the requirements of safe and reliable automatic operation, motion control has the responsibility to generate robust and stable action commands. However, due to the inevitable parameter uncertainty of the autonomous system, such as machining errors of parts, assembly clearance, and wear caused by operation, motion control based on an accurate model is not applicable. How to make the controller that independent of the system model and able to reject the disturbance of system uncertainty so as to realize the stable operation of the sweeper is an inevitable challenge.

Therefore, a heading-error-based first order linear active disturbance rejective controller (HFO-LADRC) was proposed in this paper. The active disturbance rejective controller (ADRC) is a practical control technology whose basic idea is “To reject disturbance with estimating disturbance”, which treats the internal unmodeled dynamics and external unknown disturbances as a lumped disturbance and rejects uncertain disturbance by estimating disturbance. In order to describe the lateral motion of an autonomous road sweeper, a new lateral error model has been proposed according to the kinematic relationship of the preview node, fixed onto the vehicle body, and the track node, located in the reference trajectory. The model shows that the lateral controller should eliminate lateral and heading errors through generating the front wheel steering command, that is, the system is an underactuated system. In order to eliminate the lateral error and the heading error at the same time, a new model, called a heading-error-based model, was proposed for lateral motion, and the characteristics of the Lyapunov function prove that the heading-error-based model has the ability to converge both lateral error and heading error. Since the heading-error-based model is first order, the active disturbance rejective controller (ADRC) is designed as first order and linear, and each module of the HFO-LADRC has been devised in detail, including a first-order linear tracking differentiator (LTD), a linear extended state observer (LESO), and linear state error feedback (LSEF). In addition, a typical ring scenario and a double-lane collision avoidance scenario were designed, referencing *ISO3888-2:2011*, so as to make the simulation results closer to the actual conditions. There was (1) a ring scenario consisting of two 35 m straight lanes plus two semicircles with a radius of 2.5 m and (2) a double-lane scenario consisting of a 2 m wide and 25 m long straight interval. The main wheelbase and steering ratio uncertainty are the two main factors, considering the obvious influence. Since the error of the actual wheelbase can be controlled within 0.1 m, the real wheelbase was divided into three grades, which were respectively 1.24 m, 1.34 m, and 1.44 m, as the designed wheelbase is 1.34 m. Due to the fact that the error of the actual steer ratio can be controlled within 1.0, the real steer ratio was divided into three grades as well, which were respectively 4.0, 5.0, and 6.0, as the designed steer ratio is 5.0. In addition, the steer ratio could be varied during the operation of the vehicle. Therefore, a Gaussian white noise (GWN) disturbance, which had the expectation as 5 and the mean variance as 0.25, was superimposed on the designed steer ratio to simulate the change.

The test results show that, firstly, when the wheelbase has uncertainty in the ring test scenario, the peak error of the SO-LADRC is the largest, reaching almost 1.1 m, whereas the HFO-LADRC has the lowest peak error, reaching 0.0342 m, and the peak lateral error of pure pursuit reaches 0.457 m. In addition, when the wheelbase uncertainty is 0.1 m, the lateral error change of the HFO-LADRC is less than 0.0045 m, while that of pure pursuit is 0.037 m and that of the SO-LADRC is 0.017 m; secondly, when the wheelbase has uncertainty in the double lane-changing test scenario, the peak error of the SO-LADRC is the largest, reaching almost 0.5147 m, whereas the HFO-LADRC has the lowest peak error, reaching 0.04649 m, and the peak lateral error of pure pursuit reaches 0.5094 m. In addition, when the wheelbase uncertainty is 0.1 m, the lateral error change of the HFO-LADRC is less than 0.0061 m, while that of pure pursuit is 0.023 m and that of the SO-LADRC is 0.019 m; thirdly, when the steer ratio has uncertainty in the ring test scenario, the peak error of the SO-LADRC is the largest, reaching almost 1.2 m, whereas the HFO-LADRC has the lowest peak error, reaching 0.0462 m, and the peak lateral error of pure pursuit reaches 0.556 m. In addition, when the steer ratio uncertainty is 1.0, the lateral error change of the HFO-LADRC is less than 0.016 m, while that of pure pursuit is 0.14 m and that of the SO-LADRC is 0.14 m.; finally, when the steer ratio has uncertainty in the double lane-changing test scenario, the peak error of pure pursuit is the largest, reaching almost 0.5777 m, whereas the HFO-LADRC has the lowest peak error, reaching 0.08752 m and the peak lateral error of pure pursuit reaches 0.5463 m. In addition, when the steer ratio uncertainty is 1.0, the lateral error change of the HFO-LADRC is less than 0.048 m, while that of pure pursuit is 0.12 m and that of the SO-LADRC is 0.11 m. The above data further illustrate that, both in the case of wheelbase uncertainty and in the case of steer ratio uncertainty, the HFO-LADRC has strong robustness and stability compared with typical pure pursuit and the classical SO-LADRC, both in the ring test scenario and in the double lane-changing obstacle avoidance tracking test scenario.

Future work will focus on carrying out more typical test scenarios and real vehicle tests for in-depth verification of the algorithm, and the consideration of more types of uncertainties will make the algorithm more robust and practical. Further research into the dynamic characteristics of the actuator will help in the development of functions in emergency situations, such as auto emergency braking (AEB). It is worth studying the problem of multi-system join operations, such as rejecting delay disturbance when the motion controller, environment preceptor, decision maker and trajectory planner are joint working.

## Figures and Tables

**Table 1:** Key parameters.

Parameter	Setting	Parameter	Setting	Parameter	Setting
Designed wheelbase *L*	1.34 m	Working speed *v_d_*	5.0 km/h	Preview distance *l_s_*	1.34 m
Designed Steer ratio *i*	5.0	Maximum steer angle	0.698 rad	LESO period *T_LESO_*	0.01 s
*c*_0_	0.09 *π*/*l_s_*	*c*_1_	10/*l_s_*	*c*_2_	0.1/*l_s_*
*b*_0_	−0.1 *v_d_*/*l_s_*/*L*	LSEF gain *w_c_*	0.4	LESO gain *w_o_*	4

**Table 2:** Method and real wheelbase *L*.

Label	Method	Real *L*	Label	Method	Real *L*	Label	Method	Real *L*
Pur1	Pure Pursuit	1.34 m	SOL1	SO-LADRC	1.34 m	HFO1	HFO-LADRC	1.34 m
Pur2	Pure Pursuit	1.44 m	SOL2	SO-LADRC	1.44 m	HFO2	HFO-LADRC	1.44 m
Pur3	Pure Pursuit	1.24 m	SOL3	SO-LADRC	1.24 m	HFO3	HFO-LADRC	1.24 m

**Table 3:** Method and real steer ratio *i*.

Label	Method	Real *i*	Label	Method	Real *i*	Label	Method	Real *i*
Pur1	Pure Pursuit	5.0	SOL1	SO-LADRC	5.0	HFO1	HFO-LADRC	5.0
Pur2	Pure Pursuit	6.0	SOL2	SO-LADRC	6.0	HFO2	HFO-LADRC	6.0
Pur3	Pure Pursuit	4.0	SOL3	SO-LADRC	4.0	HFO3	HFO-LADRC	4.0

**Table 4:** Method and real steer ratio *i*.

Label	Method	Real *i*	Label	Method	Real *i*	Label	Method	Real *i*
Pur1	Pure Pursuit	5.0	SOL1	SO-LADRC	5.0	HFO1	HFO-LADRC	5.0
Pur2	Pure Pursuit	GWN	SOL2	SO-LADRC	GWN	HFO2	HFO-LADRC	GWN
